# On point estimation of the abnormality of a Mahalanobis index

**DOI:** 10.1016/j.csda.2016.01.014

**Published:** 2016-07

**Authors:** Fadlalla G. Elfadaly, Paul H. Garthwaite, John R. Crawford

**Affiliations:** a Department of Mathematics and Statistics, The Open University, UK; b Department of Statistics, Faculty of Economics and Political Science, Cairo University, Egypt; c School of Psychology, King’s College, University of Aberdeen, UK

**Keywords:** Bernstein polynomials, Mahalanobis distance, Median estimator, Plug-in maximum likelihood, Quadrature approximation, Unbiased estimation

## Abstract

Mahalanobis distance may be used as a measure of the disparity between an individual’s profile of scores and the average profile of a population of controls. The degree to which the individual’s profile is unusual can then be equated to the proportion of the population who would have a larger Mahalanobis distance than the individual. Several estimators of this proportion are examined. These include plug-in maximum likelihood estimators, medians, the posterior mean from a Bayesian probability matching prior, an estimator derived from a Taylor expansion, and two forms of polynomial approximation, one based on Bernstein polynomial and one on a quadrature method. Simulations show that some estimators, including the commonly-used plug-in maximum likelihood estimators, can have substantial bias for small or moderate sample sizes. The polynomial approximations yield estimators that have low bias, with the quadrature method marginally to be preferred over Bernstein polynomials. However, the polynomial estimators sometimes yield infeasible estimates that are outside the 0–1 range. While none of the estimators are perfectly unbiased, the median estimators match their definition; in simulations their estimates of the proportion have a median error close to zero. The standard median estimator can give unrealistically small estimates (including 0) and an adjustment is proposed that ensures estimates are always credible. This latter estimator has much to recommend it when unbiasedness is not of paramount importance, while the quadrature method is recommended when bias is the dominant issue.

## Highlights

• We seek an estimate of the population proportion with extreme Mahalanobis index.
• Nine point estimates are examined in an extensive simulation study.
• Maximum likelihood estimates have substantial bias.
• Methods based on polynomial approximations give low bias, but can be out-of-range.
• An adapted median estimator that always gives sensible estimates is proposed.

## 1 Introduction

The Mahalanobis distance is frequently used in multivariate analysis as a statistical measure of distance between a vector of scores for a single case and the mean vector of the underlying population or a sample of data. It was developed by Mahalanobis (1936) as a distance measure that incorporates the correlation between different scores. See also DasGupta (1993). The Mahalanobis distance of a vector x, of say $\nu_{1}$ variables (scores), from a population mean μ is defined as

(1) $$\Delta =\sqrt{{\left(x-\mu \right)}^{\prime }\Sigma^{-1}\left(x-\mu \right)}\text{,}$$

where Σ is the population covariance matrix. The square of the Mahalanobis distance, $\Delta^{2}$, is sometimes referred to as the Mahalanobis index (Huberty and Olejnik, 2006, p. 271). If the population follows a multivariate normal distribution (MVN) and x is an observation from this same distribution, then the Mahalanobis index follows a central chi-square distribution on $\nu_{1}$ degrees of freedom. In this paper, interest focuses on estimating P, the proportion of the population that gives a more unusual Mahalanobis index than ${\left(x^{*}-\mu \right)}^{\prime }\Sigma^{-1}\left(x^{*}-\mu \right)$ where $x^{*}$ is a specified vector, under the assumption that the population distribution is a MVN distribution. That is

(2) $$P=\mathrm{Pr}\left\{{\left(x-\mu \right)}^{\prime }\Sigma^{-1}\left(x-\mu \right)>{\left(x^{*}-\mu \right)}^{\prime }\Sigma^{-1}\left(x^{*}-\mu \right)\right\}\text{,}$$

where x∼MVN(μ,Σ). For example, $x^{*}$ might be a patient’s profile from a set of medical tests, when P would be the proportion of the population with a profile that is more unusual than that of the patient.

The corresponding Mahalanobis distance in a sample, of say n observations, is defined as

(3) $$\overset{˜}{D}=\sqrt{{\left(x-\overset{¯}{x}\right)}^{\prime }S^{-1}\left(x-\overset{¯}{x}\right)}\text{,}$$

where $\overset{¯}{x}$ and S are the sample mean vector and sample covariance matrix, respectively. Under the assumption that x and the sample data are from the same MVN distribution, the sample Mahalanobis index $\left({\overset{˜}{D}}^{2}\right)$ is proportional to a central F distribution with $\nu_{1}$ and $\nu_{2}\equiv n-\nu_{1}$ degrees of freedom. See, for example, Mardia et al. (1979).

We were initially motivated by the need to estimate the abnormality of a single patient’s profile in neuropsychology. The problem arises, for example, when psychologists need to assess how a patient with some brain disorder or a head injury is different from the general population or some particular subpopulation. This assessment is usually based on the patient’s scores in a set of tests that measure different traits or abilities. The abnormality of the case’s profile of scores can then be expressed in terms of the Mahalanobis index between this profile and the mean of the normative population or normative sample. The degree of abnormality is measured by

(4) P^=Pr{(x−x¯)′S−1(x−x¯)>(x∗−x¯)′S−1(x∗−x¯)},

where $x^{*}$ is the case’s profile and is treated as a fixed quantity.

A Hotelling’s $T^{2}$ significance test for testing whether the case could belong to the normative population is proposed in Huizenga et al. (2007). Their test is based on the central F distribution to which the Hotelling’s test statistic is proportional. Crawford et al. (2016) give a confidence interval for the probability (P) of getting a more extreme profile than the case. The confidence interval is based on a non-central F distribution with a non-centrality parameter that is proportional to the case’s Mahalanobis index. The confidence intervals are correct, in that their coverage levels equal the nominal confidence level exactly. In contrast, the p-value from the Hotelling’s $T^{2}$ test provides an obvious point estimator of P, but it is biased. Indeed, the problem of finding an unbiased estimator of P has not been resolved.

Here we consider a number of obvious estimators of P and propose some new, less obvious estimators. The bias and mean square error of all the estimators are compared in extensive simulations. No estimator is uniformly better than all alternatives, but a small selection of the estimators is clearly to be preferred. As well as bias and mean square error, other criteria and desirable qualities in an estimator are also considered. In this paper, no distributional assumptions are made about the source of $x^{*}$, other than when testing whether $x^{*}$ could be the profile of a member of the normative population.

The need to estimate the value of P for Mahalanobis distances does not only arise in psychology. In the literature, the commonly used estimates of P are the p-value computed from the chi-square distribution of the sample Mahalanobis index, or the p-value from the central F distribution associated with Hotelling’s $T^{2}$ test. For example, in remote sensing image analysis, Foody (2006) was interested in measuring the closeness of an image pixel to a single class centroid. For that, he used the Mahalanobis distance and converted the calculated Mahalanobis distance, of a particular image pixel from a specified class centroid, to its associated p-value from the chi-square distribution. He then interpreted the p-value as the probability of obtaining a Mahalanobis distance as extreme as that observed for a particular pixel with respect to a specified class, thus effectively equating the p-value to P.

In environmental and health science, Liu and Weng (2012) used Mahalanobis distance in public health studies to enhance the resolution of satellite imagery. They conducted a spatial–temporal analysis of West Nile Virus outbreak in Los Angeles in 2007 using sensing variables and infective mosquito surveillance records. Mahalanobis distance was used to identify and map the risk areas where habitat was suitable for infective mosquitoes. Liu and Weng (2012) calculated the distance between a vector of environmental variables and the mean vector of environmental factors at the closest locations of mosquito infections. Locations with smaller values of Mahalanobis distances indicated a more favorable habitat for the mosquitoes and hence an area of higher risk. They assumed that Mahalanobis distance follows a chi-square distribution, from which P was calculated for each map pixel. Pixels with P between 0.6 and 0.9 (0.9 and 1.0) were considered moderate risk (high risk) areas and then a risk map was produced.

In analytical chemistry, Shah and Gemperline (1990) were interested in analyzing the near-infrared reflectance spectra of raw materials. They used Mahalanobis distance as a classification technique for pattern recognition to classify new samples by comparing them to measurements of predetermined classes. Each sample was classified according to the p-value associated with its Mahalanobis distance from the class centroids. Shah and Gemperline (1990) needed to estimate the p-value for each new sample and used the chi-square distribution to estimate these probabilities. They considered samples with probability levels between (0–0.01), (0.01–0.05) or (0.05–1.0) to be nonmembers, outliers or members, respectively.

A sample Mahalanobis distance has an exact F distribution. Hence, unsurprisingly, the F distribution has also been frequently used to quantify the rarity/commonness of a Mahalanobis distance. For example, Lu et al. (2005) used the two groups Hotelling’s $T^{2}$ test for detecting differential expressions in genetic microarrays. They conducted a microarray experiment in which samples from a disease group and from a normal group were obtained. They based their test for gene differential expression on the scaled F distribution of the Hotelling’s $T^{2}$ statistic. Some other important applications of the Hotelling’s $T^{2}$ statistic, Mahalanobis distance and the associated p-values include, for example, multivariate outlier detection (e.g. Garrett, 1989; Hardin and Rocke, 2005) and multivariate quality control charts (e.g. Sullivan and Woodall, 1998; Johnson and Wichern, 2007). However, some methodological researches in causal inference argue that Mahalanobis distances do not work very well when the dimension, $\nu_{1}$, of x is greater than 8 or the normality assumption is not fulfilled. See, for example, Stuart (2010) and the references therein.

We conducted a simulation study to test the performance of the sample p-value associated with the F distribution, denoted by P^F, and that associated with the chi-square distribution, denoted by P^χ2, in estimating the probability P. Simulation results show that both are biased estimates of P. We propose some alternative estimators of P and compare them in terms of their bias and root mean square error in the simulation study. Some of the proposed estimates have much lower biases than the estimators derived from the F and chi-square distributions.

Three of the alternative point estimators of P are based on its confidence intervals. The first uses the frequentist median of the non-centrality parameter, and is denoted by P^D. The second proposed estimator uses the Bayesian median of the non-centrality parameter or its frequentist median, whichever is greater. We call it the modified median estimator and denote it by P^MD; P^D and P^MD only differ when P approaches 100%. The third estimator in this group is a Bayesian estimator; it is based on the idea of probability matching priors and is denoted by P^BY. We propose another two new estimators of P based on the mean of the non-centrality parameter of a non-central F distribution; these are denoted by P^M and P^R. Estimators derived from a Taylor expansion (P^T)–Bernstein polynomials of degree 4, 7 and 10 (P^B4, P^B7 and P^B10) and a quadrature polynomial approximation of degree 4, 7 and 10 (P^Q4, P^Q7 and P^Q10)–are also proposed and shown to be approximately unbiased in the broad range of situations examined in the simulation study.

The paper is organized as follows. In Section  2, we briefly discuss the two frequently used point estimators of P. The new twelve proposed point estimators of P are detailed in Section  3. All the fourteen point estimators are then compared in Section  4, where we present and discuss the results of the simulation study. In Section  5, we examine the behavior of each estimator at different observed values of the Mahalanobis index. We also briefly consider the median error of the estimators (rather than average error) and mean absolute error. Concluding comments are given in Section  6.

## 2 Two plug-in maximum likelihood estimators of P

We aim to derive an unbiased estimate of

(5) $$P=\mathrm{Pr}\left\{\chi_{\nu_{1}}^{2}>\lambda \right\}\text{,}$$

where $\chi_{\nu_{1}}^{2}$ is the population Mahalanobis index ${\left(x-\mu \right)}^{\prime }\Sigma^{-1}\left(x-\mu \right)$, which follows a chi-square distribution on $\nu_{1}$ degrees of freedom, and $\lambda =\Delta^{2}$ is the Mahalanobis index of the case. That is λ equals ${\left(x^{*}-\mu \right)}^{\prime }\Sigma^{-1}\left(x^{*}-\mu \right)$, where $x^{*}$ is the vector of a case’s profile of scores from $\nu_{1}$ tests. The probability P is the proportion of the population that has a profile more extreme than the case. A minimum variance unbiased estimator of λ is readily available (see Section  3.2) but obtaining an unbiased estimator of P is much harder.

Let $\overset{¯}{x}$ and Σ^ denote the maximum likelihood estimates of μ and Σ, respectively, hence Σ^=[(n−1)/n]S. Simple estimates of P can be obtained by replacing the unknown parameters in Eq. (2) with their maximum likelihood estimates. This gives our first estimator,

(6) P^F=Pr{(x−x¯)′Σ^−1(x−x¯)>(x∗−x¯)′Σ^−1(x∗−x¯)}.

It is well-known that

(7) P^F=Pr{Fν1,ν2>[ν2(n−1)ν1]T2},

where

$$\lambda_{0}={\left(x^{*}-\overset{¯}{x}\right)}^{\prime }S^{-1}\left(x^{*}-\overset{¯}{x}\right)\text{.}$$

$T^{2}=n\lambda_{0}/\left(n+1\right)$ is Hotelling’s $T^{2}$ statistic and $F_{\nu_{1},\nu_{2}}$ is a central F distribution on $\nu_{1}$ and $\nu_{2}$ degrees of freedom. Then P^F is the p-value from testing the null hypothesis that the case is a member of the control population. Consequently, it is commonly used as a point estimate of the proportion of the normative population with more extreme profiles than the case.

Another frequently used plug-in maximum likelihood estimate, denoted by P^χ2, is the p-value from the chi-square distribution. Instead of replacing μ and $\Sigma^{-1}$ by $\overset{¯}{x}$ and Σ^−1 everywhere in (2), P^χ2 is obtained by only making this replacement on the right-hand side of the inequality. Thus

(8) P^χ2=Pr{(x−μ)′Σ−1(x−μ)>(x∗−x¯)′Σ^−1(x∗−x¯)},

and we have that

(9) P^χ2=Pr{χν12>nn−1λ0}.

Simulation results in Section  4 show that P^χ2 is generally better than P^F as an estimate of P. However, both are biased and P^χ2 underestimates P in most cases, with absolute bias that is getting higher for larger values of the true parameter P.

## 3 New point estimators of P

### 3.1 Estimators derived from confidence intervals

#### 3.1.1 Classical estimator of the median

Based on the work of Reiser (2001), Crawford et al. (2016) proposed a method for constructing confidence intervals on P. The observable sample statistic $F_{0}=\left[n\;\nu_{2}/\left(n-1\right)\;\nu_{1}\right]\;\lambda_{0}$ has a non-central F distribution with $\nu_{1}$, $\nu_{2}$ degrees of freedom, respectively, and a non-centrality parameter nλ, i.e. 

(10) $$F_{0}=\left[\frac{n\;\nu_{2}}{\left(n-1\right)\;\nu_{1}}\right]\lambda_{0}∼F_{\nu_{1},\nu_{2}}\left(n\lambda \right)\text{.}$$

To construct a confidence interval for P, define $L_{\alpha }$ as the value of nλ for which $F_{0}$ is the α-quantile of $F_{\nu_{1},\nu_{2}}\left(n\lambda \right)$. Then, a 100(1−α)% confidence interval for P is given by

(11) $$\left\{1-G\left(L_{\alpha /2}/n\right),1-G\left(L_{1-\alpha /2}/n\right)\right\}\text{,}$$

where G(.) is the cdf of a chi-square distribution on $\nu_{1}$ degrees of freedom.

Using the same technique, a point estimator of P is given by its median estimate. We have that $L_{0.5}$ is the value of nλ at which $F_{0}$ is the median of the $F_{\nu_{1},\nu_{2}}\left(n\lambda \right)$ distribution. The first of our new estimators, P^D, is defined as

(12) P^D=1−G(L0.5n).

Although P^D is a biased estimator of P, simulation results in Section  4 show that it usually has a smaller bias and mean square error than P^F at all values of the true parameter P.

#### 3.1.2 Modified estimator of the median

As nλ decreases, so does the median of $F_{\nu_{1},\nu_{2}}\left(n\lambda \right)$. Since λ≥0, a lower bound on the median of $F_{\nu_{1},\nu_{2}}\left(n\lambda \right)$ is the median of $F_{\nu_{1},\nu_{2}}\left(0\right)$. ($F_{\nu_{1},\nu_{2}}\left(0\right)$ is the ordinary central F distribution on $\nu_{1}$ and $\nu_{2}$ degrees of freedom.) If $F_{0}$ is less than this lower bound, one approach is to set nλ to zero. This is the standard approach adopted in the construction of confidence intervals, where the same problem arises, as discussed in Reiser (2001). The problem arises whenever $F_{0}$ is small, even if it is above the lower bound. To illustrate, suppose $\nu_{1}=4$, $\nu_{2}=20$ and $\lambda_{0}=0.4$, so $F_{0}=2.09$. Then calculation gives P^D=99.49%. Thus when a patient’s *estimated* Mahalanobis index is 0.4, then 0.51% is the estimate of the proportion of the normal population with a smaller true Mahalanobis index than the case. However, if 0.4 were the *true* Mahalanobis distance of the case, then the actual proportion of the normal population with a smaller Mahalanobis distance than the case is calculated at 1.75% (P=0.9825), from a chi-square distribution on 4 degrees of freedom. The disparity between 0.51% and 1.75% is substantial and, moreover, intuitively one would expect uncertainty to result in P^D being less extreme than P, rather than being greater than it. As $\lambda_{0}$ decreases the situation worsens. When $\lambda_{0}=0.2$, P^D=99.99%, while P=99.53% when λ=0.2.

A pragmatic solution was proposed by Garthwaite et al. (2016). They supposed an individual’s sample Mahalanobis index was $\lambda_{0}$ and considered the question: “What proportion of the population will have a true Mahalanobis index that is bigger than this individual?” under two situations

(i) the individual is the case
(ii) the individual is a randomly chosen member of the population.

They argue that the answer in situation (i) should be no bigger than in situation (ii). They suggest that the proportion should be estimated for each situation and the smaller estimate selected. Adopting that approach, we construct another estimator, P^MD, as follows.

Let $\overset{˜}{\lambda }$ be the Mahalanobis index of a randomly selected individual from the population and let $\overset{˜}{P}$ be the proportion of the population with a larger Mahalanobis index than $\overset{˜}{\lambda }$. Then $\overset{˜}{P}$ is a random variable and, before observing $\lambda_{0}$, $\overset{˜}{P}$ has a uniform distribution over the interval (0, 1). In consequence, $\overset{˜}{\lambda }$ has a chi-square distribution on $\nu_{1}$ degrees of freedom. This chi-square distribution can be taken as the prior distribution in a Bayesian analysis in which there is a single datum, $\lambda_{0}$. Note that there is nothing arbitrary about this prior distribution; it is the distribution of $\overset{˜}{\lambda }$ because $\overset{˜}{P}∼U\left(0,1\right)$. The likelihood follows from Eq. (10) as $F_{0}=\left[n\;\nu_{2}/\left(n-1\right)\;\nu_{1}\right]\lambda_{0}|\overset{˜}{\lambda }∼F_{\nu_{1},\nu_{2}}\left(n\overset{˜}{\lambda }\right)$. We obtain the posterior distribution of λ, and compute its normalizing constant through numerical integration. A simple search procedure is used to find the posterior median of $\overset{˜}{\lambda }$, say $M_{\lambda }$. We then use $M_{\lambda }$ to enhance the median estimator P^D in (12) and propose the modified median estimator, P^MD, as

(13) P^MD=min{P^D,1−G(Mλ)}.

**Fig. 1:**
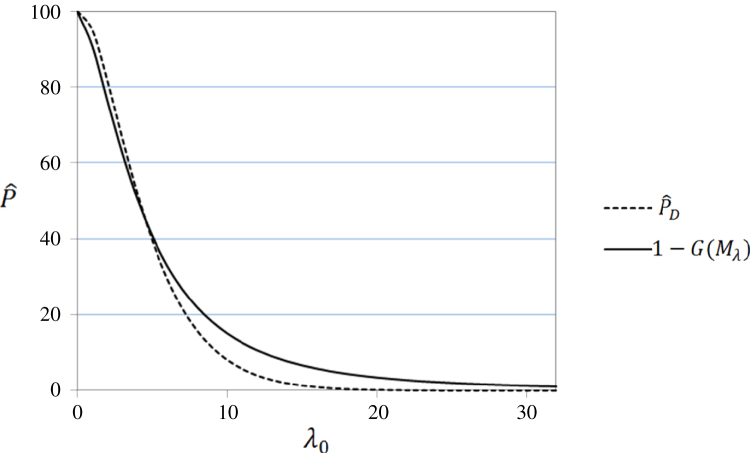
P^D and $1-G\left(M_{\lambda }\right)$ at $0\leq \lambda_{0}\leq 32$.

**Fig. 2:**
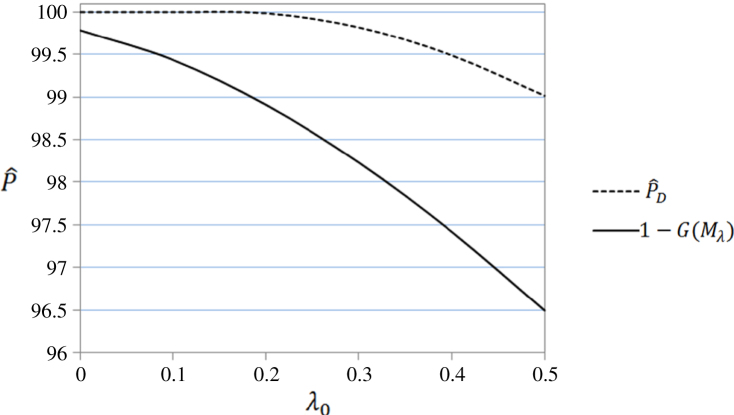
P^D and $1-G\left(M_{\lambda }\right)$ at $0\leq \lambda_{0}\leq 0.5$.

Obviously, P^MD and P^D only differ when $\lambda_{0}$ is small and then the differences are slight in absolute terms (|P^MD–P^D|), though P^D/P^MD is far from 1 for very small $\lambda_{0}$. This is illustrated, for $\nu_{1}=4$ and $\nu_{2}=20$, in Fig. 1, Fig. 2, where P^D and $1-G\left(M_{\lambda }\right)$ are plotted against $\lambda_{0}$ at different observed values of $0\leq \lambda_{0}\leq 32$ and $0\leq \lambda_{0}\leq 0.5$, respectively. As P^MD takes the lower value of P^D and $1-G\left(M_{\lambda }\right)$, Fig. 1 shows that P^D and P^MD are identical for $\lambda_{0}>4.5$, while Fig. 2 shows that, as $\lambda_{0}$ gets small, P^MD is clearly less than 100% (as common sense dictates it should be) while P^D approaches 100%.

As expected, simulation results in Section  4 show that the bias and mean square error of the modified estimator P^MD are nearly identical to those of the median estimator P^D. We recommend P^MD over P^D for use in practice to avoid the problem of getting P^D=1, as discussed above.

#### 3.1.3 Bayesian probability matching

Bayesian 100(1−α)% credible intervals quite often have the same endpoints as frequentist 100(1−α)% confidence intervals if the Bayesian intervals are based on uninformative prior distributions. Indeed, there has been substantial interest in *probability matching priors*  (Datta and Mukerjee, 2004), which are designed to give credible intervals that match confidence intervals. To construct our next estimate of P, we suppose a prior distribution has been found that gives posterior credible intervals which match the confidence intervals specified in Eq. (11). Treating the confidence intervals as exact credible intervals, they determine a posterior distribution for the proportion P. We use a sufficiently large number, say R, of one-sided credible interval limits to construct the posterior distribution. The posterior mean is then used as a point estimate, say P^BY, of P.

Specifically, we estimate the interval limit $L_{r/R}$ as the value of nλ for which $F_{0}$ is the (r/R)-quantile of $F_{\nu_{1},\nu_{2}}\left(n\lambda \right)$, for r=1,…,R. As in (11), the (r/R)-quantile of the posterior distribution of P is given by $1-G\left(L_{r/R}/n\right)$. The posterior mean P^BY is then computed as

(14) P^BY=1−∑r=1RG(Lr/R/n)R.

In practice, we take R=500, so that we use the quantiles (0.002,0.004,…,0.998) which is a sufficiently fine partition for our purpose and is not expensive in computing time.

Based on simulation results in Section  4, the estimate P^BY is a badly biased estimate of P. This result illustrates an important fact: while posterior distributions obtained from exact probability matching priors will (by design) give interval estimates with good frequentist properties, the posterior mean may be far from meeting the frequentist definition of unbiasedness.

### 3.2 Estimators based on the mean of λ

Our next proposed estimators of P are based on the estimated mean value of λ, say $\overset{¯}{\lambda }$. If $F_{0}$ is given by Eq. (10), then

(15) $$n\overset{¯}{\lambda }=\frac{\nu_{1}\left(\nu_{2}-2\right)}{\nu_{2}}F_{0}-\nu_{1}$$

is the uniformly minimum variance unbiased estimator of the non-centrality parameter of the non-central F distribution $F_{\nu_{1},\nu_{2}}\left(n\lambda \right)$. However, it is well-known that this is not always positive and is therefore inadmissible. See, for example, Johnson et al. (1995). To avoid a negative estimate of λ, put

(16) λ^=Max{1n[ν1(ν2−2)ν2F0−ν1],0}.

Using λ^, we propose the estimator P^M of P as

(17) P^M=1−G(λ^).

Unfortunately, based on the simulation results in Section  4, P^M can have marked bias as an estimator of P.

The estimator λ^ in (16) is also inadmissible (Chow, 1987), but Rukhin (1993) showed that, for $\nu_{2}>4$,

(18) $$\overset{˜}{\lambda }=\frac{1}{n}\left[\frac{\nu_{1}\left(\nu_{2}-4\right)}{\nu_{2}}F_{0}\right]$$

is an admissible estimator of λ. We base our next estimate, P^R, of P on $\overset{˜}{\lambda }$ and put

(19) P^R=1−G(λ˜).

However, as illustrated in Section  4, P^M is generally better than P^R in terms of bias and mean square error.

### 3.3 An estimator based on a Taylor expansion

We expand the cdf of the chi-square distribution G(X) about λ as

(20) $$G\left(X\right)≃G\left(\lambda \right)+\left(X-\lambda \right)g\left(\lambda \right)+\frac{{\left(X-\lambda \right)}^{2}}{2}g^{\prime }\left(\lambda \right)\text{,}$$

where g(.) is the pdf of a chi-square distribution with $\nu_{1}$ degrees of freedom. We set X equal to $\overset{¯}{\lambda }$ in Eq. (15) and take the expected value of both sides of (20). This gives

(21) $$E\left\{G\left(\overset{¯}{\lambda }\right)\right\}≃G\left(\lambda \right)+\frac{\mathrm{Var}\left(\overset{¯}{\lambda }\right)}{2}g^{\prime }\left(\lambda \right)\text{,}$$

where, from the variance of $F_{0}$ with $\nu_{2}>4$, $\mathrm{Var}\left(\overset{¯}{\lambda }\right)$ is given by

(22) $$\text{Var}\left(\overset{¯}{\lambda }\right)≃v_{0}+v_{1}\;\lambda +v_{2}\;\lambda^{2}\text{,}$$

with $v_{0}=2\nu_{1}\left(\nu_{1}+\nu_{2}-2\right)/\left(n^{2}\left(\nu_{2}-4\right)\right)$, $v_{1}=4\left(\nu_{1}+\nu_{2}-2\right)/\left(n\left(\nu_{2}-4\right)\right)$ and $v_{2}=2/\left(\nu_{2}-4\right)$. (As defined earlier, $F_{0}∼F_{\nu_{1},\nu_{2}}\left(n\lambda \right)$.)

In the light of (21), to obtain an approximately unbiased estimate of G(λ) to the second order, it seems natural to base an estimate on $\lambda^{\#}$ say, such that

(23) $$G\left(\lambda^{\#}\right)-G\left(\overset{¯}{\lambda }\right)=-\frac{\text{Var}\left(\overset{¯}{\lambda }\right)}{2}g^{\prime }\left(\lambda \right)\text{.}$$

We start with the case where $\overset{¯}{\lambda }$ is greater than the mode of the chi-square distribution. In this case $g^{\prime }\left(\lambda \right)$ is negative, $\lambda^{\#}>\overset{¯}{\lambda }$ and we can write

(24) $$G\left(\lambda^{\#}\right)-G\left(\overset{¯}{\lambda }\right)=\left(\lambda^{\#}-\overset{¯}{\lambda }\right)g\left(\xi \right)\text{,}$$

for some $\xi \in \left(\overset{¯}{\lambda },\lambda^{\#}\right)$. From (23), (24) we have

(25) $$\lambda^{\#}-\overset{¯}{\lambda }=-\frac{\text{Var}\left(\overset{¯}{\lambda }\right)}{2}\frac{g^{\prime }\left(\lambda \right)}{g\left(\xi \right)}\text{.}$$

We define another estimate, $\lambda^{*}$, by replacing ξ in (24) with λ:

(26) $$\lambda^{*}-\overset{¯}{\lambda }=-\frac{\text{Var}\left(\overset{¯}{\lambda }\right)}{2}\frac{g^{\prime }\left(\lambda \right)}{g\left(\lambda \right)}\text{.}$$

Suppose $\left|\overset{¯}{\lambda }-\lambda \right|$ is large relative to $\left|\overset{¯}{\lambda }-\lambda^{\#}\right|$. If $\lambda >\lambda^{\#}$, then $\lambda^{*}>\lambda^{\#}$ and $\lambda^{*}$ will be better than $\lambda^{\#}$ as an estimate of λ. If $\lambda <\overset{¯}{\lambda }$, then $\lambda^{*}<\lambda^{\#}$ and $\lambda^{*}$ will again be better than $\lambda^{\#}$ as an estimate of λ. On the other hand, supposing that $\left|\overset{¯}{\lambda }-\lambda \right|$ is small relative to $\left|\overset{¯}{\lambda }-\lambda^{\#}\right|$, then g(ξ)≃g(λ) and $\lambda^{*}≃\lambda^{\#}$. The consequence is that $\lambda^{*}$ defined in (26) is expected to be better than $\lambda^{\#}$, in terms of the mean square error, as an estimate of λ. The other case in which $\overset{¯}{\lambda }$ is less than or equal to the mode of the chi-square distribution can be treated similarly.

It remains now to estimate the right hand side of (26). We find an unbiased estimate, say Var^(λ¯), of $\text{Var}\left(\overset{¯}{\lambda }\right)$ expressed as

(27) Var^(λ¯)=u0+u1λ¯+u2λ¯2,

where $u_{0}$, $u_{1}$ and $u_{2}$ are chosen such that E{Var^(λ¯)}=Var(λ¯). Specifically, equating the corresponding coefficients of λ in E{Var^(λ¯)} to those in (22), we get $u_{0}=2\nu_{1}\left(\nu_{1}+\nu_{2}-2\right)/\left(n^{2}\left(\nu_{2}-2\right)\right)$, $u_{1}=4\left(\nu_{1}+\nu_{2}-2\right)/\left(n\left(\nu_{2}-2\right)\right)$ and $u_{2}=2/\left(\nu_{2}-2\right)$. It is straightforward to show that

(28) $$\frac{g^{\prime }\left(\lambda \right)}{g\left(\lambda \right)}=\frac{1}{\lambda }\left(\frac{\nu_{1}}{2}-1\right)-\frac{1}{2}\text{.}$$

However, no simple unbiased estimate can be found for 1/λ, instead, we estimate it as $1/\overset{¯}{\lambda }$. The estimator $\lambda^{*}$ is finally expressed as

(29) λ∗=λ¯−Var^(λ¯)2[1λ¯(ν12−1)−12].

Using this Taylor based estimate, our proposed approximately unbiased estimate P^T of P is given by

(30) P^T=1−G(λ∗).

Simulation results show that P^T is usually one of the better estimates of P. More information is given in Section  4.

### 3.4 Estimators based on polynomial approximations

None of the point estimators we have proposed so far attain approximate unbiasedness uniformly for all values of P. This motivates another set of point estimators that are approximately unbiased uniformly for all P. Using polynomial approximations, we aim to base the proposed estimator of P in this section on a good global estimate of the non-centrality parameter λ. This means that in searching for an approximately unbiased estimate of P, we cover wide areas of the chi-square cdf and do not locally search around some estimate of λ, as was proposed in Section  3.3 when using the Taylor expansion. In principle, estimates based on global approximation of the cdf should prove better, in terms of bias, than an estimate based on a local approximation.

We introduce a set of unbiased estimates of P, denoted for now by P^P, which are based on approximating the probability P in (5) as a polynomial function of degree r in λ. From Weierstrass’s Theorem, any function of a variable, λ say, can be approximated by a polynomial of λ, provided the function satisfies weak regularity conditions. Now $P=\mathrm{Pr}\left(\chi_{\nu_{1}}^{2}>\lambda \right)$ is a function of λ that meets these regularity conditions, so we may put

(31) $$P=\mathrm{Pr}\left(\chi_{\nu_{1}}^{2}>\lambda \right)≃\overset{r}{\underset{i=0}{\sum }}a_{i}\;\lambda^{i}\text{.}$$

The coefficients $a_{i}\;\left(i=0,\ldots ,r\right)$ are known functions in $\nu_{1}$ (see below).

The key to exploiting Eq. (31) is that the moments of $F_{0}$ are also polynomials in λ. Specifically, with $F_{0}$ defined by Eq. (10), the ith moment, $E\left(F_{0}^{i}\right)$, is a polynomial of λ of degree i. Writing P in the polynomial form (31), it can therefore be estimated by another polynomial in $F_{0}$ as follows

(32) P^P=∑i=0rbiF0i,

where the coefficients $b_{i}\;\left(i=0,\ldots ,r\right)$ are determined such that

(33) $$\overset{r}{\underset{i=0}{\sum }}b_{i}\;E\left(F_{0}^{i}\right)=\overset{r}{\underset{i=0}{\sum }}a_{i}\;\lambda^{i}\text{.}$$

This ensures the approximate unbiasedness of P^P in estimating P. The coefficients $b_{i}\;\left(i=0,\ldots ,r\right)$ can be obtained by equating the coefficients of the corresponding terms of the polynomials on the two sides of (33). To do that, we used the computer algebraic system Maple 16.

Although this computer algebraic system does not give explicit symbolic formulas for the raw moments of a non-central F distribution without using special functions, it can efficiently give simple explicit forms of the raw moments of a non-central chi-square distribution up to any required order r. The former can then be obtained from the latter by using the following straightforward relationship between the corresponding raw moments of the two distributions (Bain, 1969):

(34) $$\mu_{i}^{\prime }\left[F_{\nu_{1},\nu_{2}}\left(\lambda \right)\right]=\mu_{i}^{\prime }\left[\chi_{\nu_{1}}^{2}\left(\lambda \right)\right]\;\frac{\Gamma \left(\nu_{2}/2-i\right)\;{\left(\nu_{2}\right)}^{i}}{\Gamma \left(\nu_{2}/2\right)\;{\left(2\nu_{1}\right)}^{i}}\text{,}$$

where $\mu_{r}^{\prime }\left[.\right]$ is the ith raw moment.

It has to be noted here that the ith raw moment of a non-central F distribution is finite only for $\nu_{2}>2i$. This puts a constraint on the valid number r of polynomial terms to be used in the proposed approximation. If n is the size of the control sample, then r must be strictly less than $\left(n-\nu_{1}\right)/2$.

Now, to apply the approach outlined in Eqs. (31), (32), (33), it remains to find a suitable polynomial approximation to be used in (31). We use two different approximations, the first is based on Bernstein polynomials while the second is a quadrature polynomial approximation.

#### 3.4.1 Bernstein polynomials approximation

From Weierstrass’s Theorem, any continuous real valued function f(x) defined on a closed interval [a,b] can be approximated by a polynomial function. See, for example, Lorentz (1986). In 1912, Bernstein gave a simple probabilistic constructive proof for Weierstrass’s Theorem by introducing the Bernstein polynomials $B_{r}\left(f;x\right)$ as a series of polynomials that converge uniformly to any continuous bounded function f(x) on the closed interval [0,1] as r→∞. See, for example, Chapter (7) in Phillips (2003). The rth Bernstein polynomial $B_{r}\left(f;x\right)$ for f(x) is defined as:

(35) Br(f;x)=∑i=0r(ri)xi(1−x)r−if(i/r).

The polynomial function $B_{r}\left(f;x\right)$ uniformly approximates f(x) on [0,1] in the sense that $\lim_{r\to \infty }\sup_{0\leq x\leq 1}\left|B_{r}\left(f;x\right)-f\left(x\right)\right|=0$ (e.g. Theorem 1, Section VII.2 in Feller, 1965).

We use Bernstein polynomials to obtain a polynomial approximation for the chi-square cdf to be used in (31). Although the domain of the chi-square cdf is [0,∞), we use an affine transformation x=(λ−a)/(b−a), for any two arbitrary values a and b, so as to work on the [0, 1] interval. The two end-points a and b are initially chosen such that the probability of getting a sample value of the non-centrality parameter λ outside the interval [a,b] is fairly negligible. Therefore, we initially take $a=L_{0.999}/n$ and $b=L_{0.001}/n$ where, as before, $L_{\alpha }$ is the value of nλ for which $F_{0}$ is the α-quantile of $F_{\nu_{1},\nu_{2}}\left(n\lambda \right)$. As will be shown at the end of Section  3.4, the accuracy of the polynomial approximation is influenced by the choice of a and b. For extremely large values of b, say above the 0.9999-quantile of the chi-square distribution, polynomial functions of small degree r are not guaranteed to give a good approximation. Also, accuracy is greatly enhanced if a is chosen to be just below the sample median value $L_{0.5}/n$ of λ. Hence, as a rule of thumb, if $L_{0.5}/n$ is greater than the mode of the chi-square distribution, our final choice of a is $a=0.99\left(L_{0.5}/n\right)$.

We then approximate P in (31) by its rth Bernstein polynomial in λ of the form

(36) Br(P;λ)=1−∑i=0r(ri)(λ−ab−a)i(b−λb−a)r−iG(a+(b−a)ir).

Clearly, the above expression of $B_{r}\left(P;\lambda \right)$ is a polynomial of degree r in λ, and we denote its coefficients by $a_{i}\;\left(i=0,\ldots ,r\right)$. The explicit form of these coefficients was obtained using the computer algebraic system Maple 16. The coefficients of λ on the left hand side of (33) are equated to their corresponding coefficients in the Bernstein polynomial approximation (36) so as to obtain the values of $b_{i}$ and, hence, P^P in Eq. (32). In this paper, we obtain the estimate P^P for r=4, 7 and 10, and denote it by P^B4, P^B7 and P^B10, respectively.

#### 3.4.2 Quadrature polynomial approximation

We adopt the quadrature formula of Sahai et al. (2004) to obtain another polynomial approximation for P. This quadrature formula gives polynomial approximations to the integration of real valued continuous functions defined on the closed interval [0, 1]. Specifically, a function f(x) on [0, 1] is approximated by a polynomial in x of degree r as

(37) Q(x)=∑i=0r(rxi)(r(1−x)r−i)f(xi),

where $x_{i}=i/r\;\left(i=0,\ldots ,r\right)$ partition the interval [0, 1] into r equal segments. The polynomial function Q(x) can then be easily integrated over any sub-interval in [0, 1]. It has been shown empirically that the approximation has good accuracy even when r is small. For example, it was used by Richard et al. (2010) to obtain an efficient polynomial approximation of degree 9 to the normal distribution function and its inverse function.

Here, we apply the quadrature formula to approximate the density function of a chi-square distribution on $\nu_{1}$ degrees of freedom with a polynomial of degree r−1, which then yields the approximation in (31) after a straightforward symbolic integration of the polynomial.

To work on the [0, 1] interval, we adopt the approach discussed in Section  3.4.1 for choosing the two end-points a and b, with the same affine transformation x=(λ−a)/(b−a). The probability P can now be approximated as

(38) PQ=1−Pr(χν12<λ)≃1−G(a)−∫x=0λ−ab−a{∑i=0r−1((r−1)xi)((r−1)(1−x)r−i−1)(b−a)g[a+ir−1(b−a)]}dx,

where g[.] is the density function of a chi-square random variable with $\nu_{1}$ degrees of freedom.

The coefficients of λ in the expression of $P_{Q}$ in (38) above are again denoted by $a_{i}\;\left(i=0,\ldots ,r\right)$, with their explicit forms obtained using Maple 16. The coefficients of λ in the left hand side of (33) are equated to their corresponding coefficients in the quadrature polynomial approximation $P_{Q}$ in (38) so as to obtain $b_{i}\;\left(i=0,\ldots ,r\right)$. This gives another form of P^P (Eq. (32)). Here we determine it for r=4, 7 and 10, and denote the resulting estimators by P^Q4, P^Q7 and P^Q10, respectively. Simulation results show that these estimators are usually marginally better than those based on Bernstein polynomials.

**Fig. 3:**
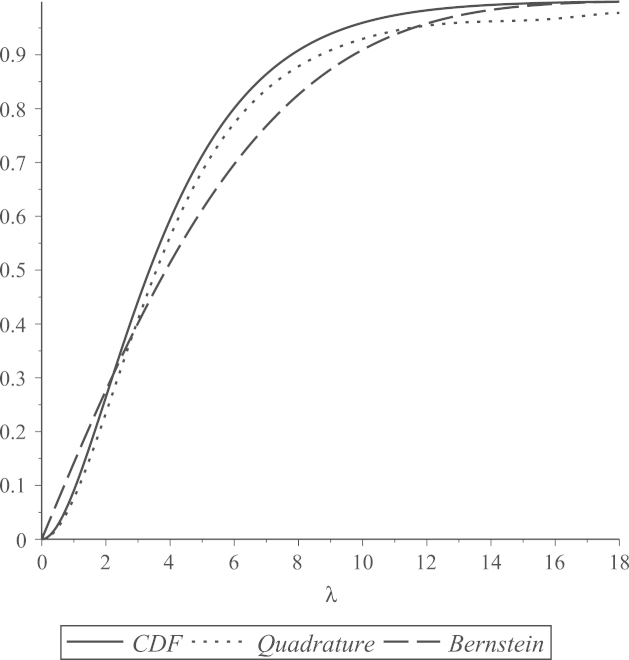
[a,b]=[0,18].

**Fig. 4:**
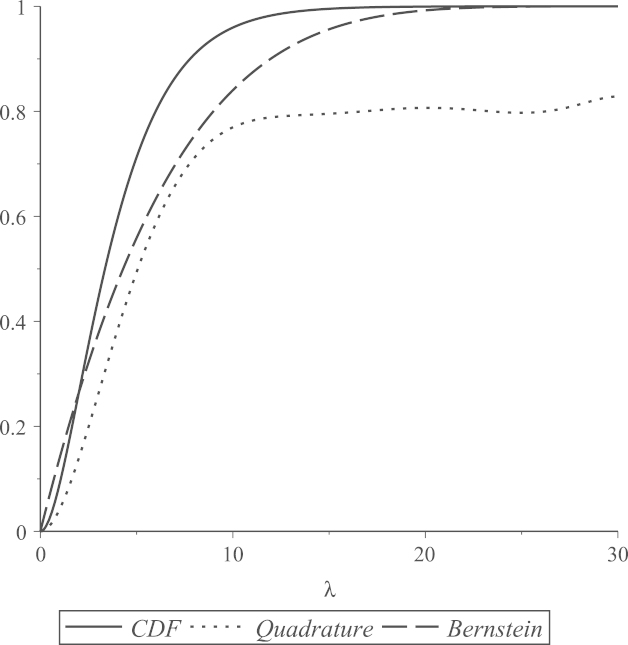
[a,b]=[0,30].

**Fig. 5:**
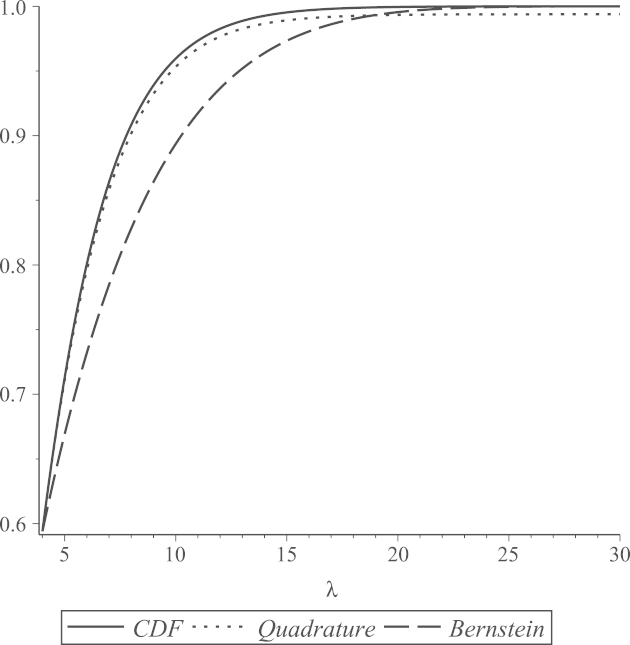
[a,b]=[4,30].

In general, polynomial functions give approximations that are accurate only on specific intervals of the domain of the underlying approximated function. The accuracy of the polynomial approximations that we use is highly related to the values selected as the two interval end-points, a and b. Fig. 3, Fig. 4, Fig. 5 show Bernstein and quadrature polynomial approximations for three choices of [a,b]. The cdf of a chi-square distribution on degrees of freedom $\nu_{1}=4$ is plotted together with its Bernstein approximation and quadrature polynomial approximation, each of degree r=7.

Fig. 3 shows that both polynomial approximations give good accuracy when [a,b] is the rather short interval [0, 18] of the cdf domain. The value b=18 is near the boundary of plausible values for a $\chi_{4}^{2}$ variate, as 18 is the 0.999 quantile of a $\chi_{4}^{2}$ distribution. For extremely large values of b, neither polynomial approximation of degree 7 is expected to attain good accuracy. This can be seen in Fig. 4, where b=30. For the same extreme value of b=30, if a is above the mode of this chi-square distribution (i.e.  a>2), remarkably better accuracy is obtained, especially for the quadrature approximation. This is shown in Fig. 5, where a=4 and b=30. This argument motivates our choice of a and b that was discussed in Section  3.4.1, as the behavior of both polynomial approximations tends to be the same for all values of $\nu_{1}$. As seen in Fig. 3 and Fig. 5, the Bernstein polynomial approximation can be more accurate than the quadrature polynomial approximation for values near b, but the quadrature approximation overall attains better accuracy over the whole interval [a,b].

## 4 Simulation results

In practice, we expect $\nu_{1}$, the number of test scores on each individual, to be small, while $n\;\left(n=\nu_{2}+\nu_{1}\right)$, the number of people in the control sample, may be large. Therefore, we conducted a simulation study that examined combinations of $\nu_{1}=2$, 4 and 8, with $\nu_{2}=10$, 20 and 80. Results of some other combinations are available on request from the authors. Based on N=100,000 samples for each combination, we tested the performance of each of the proposed estimators in terms of their average bias, ∑i=1N(P^i−P)/N, and the root of mean square error, ∑i=1N(P^i−P)2/N, denoted by SE (standard error) in tables.

Mahalanobis distance is invariant under affine transformations of location and scale parameters. Since all the methods proposed in this paper depend only on the sample Mahalanobis index, $\lambda_{0}$, we chose, without loss of generality, to set the population mean (μ) equal to 0 and the population variance (Σ) equal to the identity matrix $I_{\nu_{1}}$. The true values of P that we examined were 1%, 2.5%, 5%, 10%, 20% and 40%. These true probabilities were attained from Eqs. (2), (5) by choosing each corresponding case’s profile of scores, $x^{*}$, as a vector of equal elements. We also examined some cases where the scores in the profile, $x^{*}$, are not necessarily equal to each other. But these cases gave almost identical results to those of the profiles with equal scores. We therefore give simulation results only for profiles with equal scores.

Table 1 shows the simulation results for $\nu_{1}=2$ and 4, and $\nu_{2}=10$. When $\nu_{1}=2$, P^χ2, P^Q4 and P^T are the best three estimators in terms of the bias and root mean square error. P^χ2 is slightly better than P^Q4 and P^T up to P=10%, but for P=20% and 40%, the estimators P^Q4 and P^T are remarkably better than P^χ2 in terms of their bias, with P^Q4 being the best. At $\nu_{1}=4$, the table shows that P^χ2, P^Q4 and P^T are again the best three estimators, with P^Q4 showing less bias than P^χ2 for true values of P of 10% or more. This suggests that, at small values of $\nu_{2}$, the best two competitor estimates are P^χ2 and P^Q4, where the former is doing better at smaller values of the true probability P.

An important point from Table 1 is the cautionary message that each of the methods shows noticeable bias for some values of P at some combinations of $\nu_{1}$ and $\nu_{2}$. Four methods perform particularly poorly: P^F, P^BY, P^M, P^R.

Table 2 shows the simulation results at $\nu_{1}=2$, 4 and 8, and $\nu_{2}=20$. For all listed values of $\nu_{1}$, the three estimates P^χ2, P^Q4 and P^Q7 are doing better than the others. For small values of P, the bias of both P^Q4 and P^Q7 is generally less than that of P^χ2, while the root mean square error of P^χ2 is always less than those of P^Q4 and P^Q7. Comparing P^Q4 to P^Q7, it can be seen in Table 2 that P^Q7 is better than P^Q4 in terms of bias, although the root mean square error of P^Q4 is slightly greater than that of P^Q7 for all values of the true probability except P=1%. This suggests that P^Q7 is the best estimate at $\nu_{2}=20$ as it has rather small values of both bias and root mean square error for all values of the true probability P.

Simulation results for $\nu_{2}=80$ ($\nu_{1}=2$, 4 and 8) are presented in Table 3. This value of $\nu_{2}$ is large enough for the estimators P^Q10 and P^B10 to be computed. All estimators are doing well at this very large sample size and all have similar bias and root mean square error. However, the estimators based on polynomial approximations are still slightly better than the others. Specifically, the quadrature based estimators P^Q4, P^Q7 and P^Q10 have very low bias as shown in Table 3, with the bias of P^Q10 always less than or equal to those of P^Q4 and P^Q7.

## 5 Feasibility of estimates and absolute error

### 5.1 Ranges of estimates

The simulations in Section  4 show that the estimators based on polynomial approximations performed well, in that they had the minimum bias among the reported estimators. However, this does not mean that the estimates they produce are always sensible. Specifically, when $\lambda_{0}$ is very small they can give estimates of the proportion that are greater than 100%, and when $\lambda_{0}$ is very big they can give estimates that are less than zero. This problem comes to light by studying the behavior of the proposed estimators at different values in the domain of $\lambda_{0}$.

**Fig. 6:**
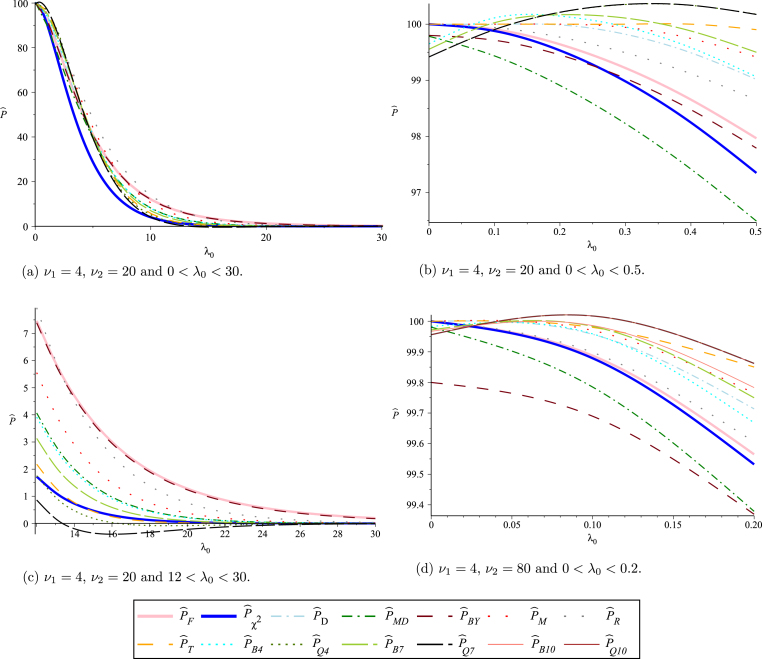
Estimates of P given by different methods for various combinations of $\nu_{1}$ and $\nu_{2}$.

For example, at $\nu_{1}=4$ and $\nu_{2}=24$, Fig. 6(a) shows that the twelve estimators all have similar patterns for $0<\lambda_{0}<30$. But a closer look at the part of the domain where $0<\lambda_{0}<0.5$ (Fig. 6(b)) reveals that P^B4, P^B7, P^Q4 and P^Q7 are not monotonically decreasing with $\lambda_{0}$ and they exceed 100% at some values of $\lambda_{0}$. With the same degrees of freedom, another problem appears in Fig. 6(c), where both P^Q4 and P^Q7 are below zero for some values of $12<\lambda_{0}<30$.

Similar problems appear at large sample sizes as well. For example, at $\nu_{1}=4$ and $\nu_{2}=80$, P^Q4 is slightly below zero for some values of $22<\lambda_{0}<32$ and as shown in Fig. 6(d), P^Q4, P^Q7 and P^Q10 all exceed 100% for some values of $0<\lambda_{0}<0.2$. The problem does not arise with other estimators—estimates are always in the range 0%–100% for P^F, P^χ2, P^D, P^MD, P^BY, P^M, P^R and P^T. However, as Fig. 6(b) shows, the estimate of P approaches 100% as $\lambda_{0}$ approaches 0. This is clearly unrealistic as the case’s value $x^{*}$ will not equal the population mean μ, even if $x^{*}$ equals the sample mean $\overset{¯}{x}$. As in Section  3.1.2, a pragmatic approach is to treat the case’s profile as that of a randomly chosen control when the case’s profile seems nearer to μ than would be expected of a control’s profile.

### 5.2 Performances as measured by absolute error

In Section  4, mean square error and average error (bias) were used to evaluate the performance of the various estimators considered in this paper. Alternative evaluation criteria include average absolute error (AAE=∑i=1N|P^i−P|/N) and median error (ME  =  median(P^1−P,…,P^N−P)). Here, we briefly examine the performance of our estimators under these criteria. In theory, the median estimator P^D should give a median error of 0 and have a lower AAE than other estimators whose median error is small. Its closely related estimator, P^MD, should also perform well.

Results for $\nu_{1}=8$ and $\nu_{2}=40$ are presented in Table 4. It can be seen that the median error in estimating P is 0.0 for both P^D and P^MD for each of the tabulated values of P. In contrast, the median error of every other estimator is never 0.0 except for P^B10 when P=40%; otherwise the median error of the other estimators is typically quite marked.

A fuller examination of the median errors given by P^D and P^MD is provided in Table 5, where results are given for these estimators for all the combinations of $\nu_{1}$ and $\nu_{2}$ that were considered in Table 1, Table 2, Table 3. The two estimators give identical median error for every combination and that error is very small in every case. Hence, if we want an estimator that has very small median error, then both P^D and P^MD can fill that role. The average absolute error is marginally better with the P^MD estimator, but the differences are very slight. However, P^MD is the preferable estimator because it will not give unrealistic estimates of P, while P^D will sometimes estimate P as 100% when that is not a credible estimate. Consequently, if a point estimator of P is required, one reasonable choice is to give P^MD as the estimator and say that it gives small median error without making any claim about its bias (average error).

## 6 Concluding comments

The task that motivated this paper seemed straightforward: find a good point estimator of the abnormality of a Mahalanobis index. The answer is less straightforward, as the best choice of estimator will depend on the purpose for which the estimator is required. The following summarizes our findings.

1. The most common criteria used to choose an estimator are bias and mean square error; the minimum variance unbiased estimator is often the preferred estimator if such an estimator can be found. Under these criteria the best estimators are those based on a quadrature polynomial approximation, P^Q4, P^Q7 and P^Q10, provided occasional negative estimates are not a problem. (The negative estimates would presumably be set to 0.) Only P^Q4 can be used for $\nu_{2}=10$; P^Q7 is best for $\nu_{2}=20$; P^Q10 and P^Q7 are marginally the best (P^Q4 is almost as good) for $\nu_{2}=80$.
2. If mean square error is to be minimized and bias is unimportant, then P^χ2 is the best estimator, but it displays substantial bias even when $\nu_{2}$ is large.
3. Sometimes, an estimate of P is to be used as an input into further analysis. Commonly though, an estimate of P is to be communicated to others (perhaps in a journal paper or a technical report) and then a good descriptive statistic is required. In that context, the best estimator would seem to be the modified median estimator, P^MD. It should be referred to as the median estimator as that is accurate: it is designed to give low median bias rather than low average bias and, indeed, its median bias is very low. It is preferable to the median estimate (P^D) because it always gives sensible estimates while P^D sometimes gives estimates that are unrealistically small when judged by common sense.
Based on our simulation results, we recommend that P^MD should generally be used as the point estimator of P. However, if unbiasedness of the required estimate is crucially important we recommend that P^Q4 should be used for $\nu_{2}<20$ and P^Q7 should be used for $\nu_{2}\geq 20$. Out-of-range values of these two estimators need to be artificially constrained so as not to lie outside the interval [0, 1].
This work is part of an on-going project that develops statistical methods for analyzing single patient data, and these recommendations are implemented in software for making inferences from Mahalanobis distance about the abnormality of an individual’s test score profile. Previous methods that we have developed are well-used by neuropsychologists (see, for example, papers that cite Crawford and Garthwaite, 2002, Crawford and Garthwaite, 2005, Crawford and Garthwaite, 2007) so it is likely that the recommendations will influence practice. The work in this paper makes these recommendations well-informed.

## Figures and Tables

**Table 1:** Bias and root mean square error (SE) of the proposed estimates of P at $\nu_{2}=10$.

$\nu_{1}$		1%		2.5%		5%		10%		20%		40%	
		Bias	SE	Bias	SE	Bias	SE	Bias	SE	Bias	SE	Bias	SE
2	P^F	4.6	7.1	5.9	9.4	6.8	11.5	7.2	14.0	6.2	16.5	2.3	18.7
	P^χ2	0.3	2.5	0.1	4.1	−0.5	6.1	−1.8	9.3	−4.5	14.2	−9.0	21.2
	P^D	1.8	4.6	2.5	6.9	3.0	9.3	3.3	12.5	2.7	16.5	0.3	20.6
	P^MD	1.8	4.6	2.5	6.9	3.0	9.3	3.3	12.4	2.4	16.0	−1.0	19.1
	P^BY	4.5	6.9	5.5	8.9	6.4	11.1	7.2	13.9	5.4	16.1	2.1	19.1
	P^M	3.4	6.8	4.9	9.7	6.4	12.7	7.8	16.3	8.6	20.1	7.5	22.8
	P^R	6.8	10.6	9.4	14.2	11.8	17.5	14.0	20.9	15.1	23.5	12.5	22.8
	P^T	0.8	3.9	1.2	6.2	1.6	9.1	2.0	13.0	2.1	18.1	1.7	22.9
	P^B4	2.0	5.0	2.8	7.4	3.6	10.1	4.2	13.7	4.3	18.1	3.2	22.1
	P^Q4	0.9	3.8	1.3	6.2	1.6	8.9	1.9	12.8	1.9	17.8	1.3	22.8
4	P^F	7.0	10.5	8.6	13.2	9.7	15.4	10.1	17.6	8.4	19.4	2.5	20.6
	P^χ2	−0.2	2.2	−0.9	3.7	−2.1	5.7	−4.7	9.3	−9.6	15.7	−18.1	26.2
	P^D	2.8	7.1	3.9	9.9	4.7	12.8	5.1	16.2	4.1	20.2	0.3	23.9
	P^MD	2.8	7.0	3.9	9.8	4.7	12.5	4.9	15.7	3.4	18.7	−2.1	21.1
	P^BY	7.2	10.7	8.4	13.2	9.7	15.3	9.4	17.4	8.2	19.4	2.9	20.5
	P^M	5.3	10.4	7.4	14.1	9.2	17.5	10.8	21.2	11.2	24.7	8.4	26.1
	P^R	10.6	16.4	14.0	20.9	17.0	24.7	19.7	28.3	20.6	30.5	16.9	28.2
	P^T	1.6	6.4	2.4	9.7	3.3	13.4	4.1	18.0	4.5	23.7	3.5	28.4
	P^B4	3.1	7.6	4.3	10.7	5.4	13.8	6.1	17.7	5.9	22.1	3.1	25.5
	P^Q4	1.4	5.8	2.0	8.8	2.6	12.2	2.9	16.6	2.7	22.5	1.7	28.7

**Table 2:** Bias and root mean square error (SE) of the proposed estimates of P at $\nu_{2}=20$.

$\nu_{1}$		1%		2.5%		5%		10%		20%		40%	
		Bias	SE	Bias	SE	Bias	SE	Bias	SE	Bias	SE	Bias	SE
2	P^F	2.2	3.8	3.1	5.6	3.7	7.4	4.1	9.6	3.5	12.2	1.2	14.6
	P^χ2	0.2	1.8	0.1	3.1	−0.3	4.8	−1.1	7.4	−2.6	11.2	−5.1	15.9
	P^D	0.9	2.6	1.3	4.3	1.6	6.2	1.8	8.8	1.3	12.1	0.0	15.5
	P^MD	0.9	2.6	1.4	4.3	1.7	6.2	1.8	8.8	1.4	12.1	−0.3	15.0
	P^BY	2.2	3.8	3.2	5.8	3.8	7.3	4.1	9.4	3.4	12.0	1.5	14.7
	P^M	1.5	3.3	2.3	5.3	3.0	7.4	3.8	10.2	4.2	13.5	3.6	16.3
	P^R	2.4	4.4	3.7	6.6	4.9	8.9	6.2	11.8	6.8	14.5	5.6	16.0
	P^T	0.3	2.1	0.4	3.8	0.5	5.8	0.6	8.8	0.5	12.8	0.4	16.5
	P^B4	0.9	2.6	1.3	4.4	1.7	6.4	2.0	9.2	1.9	12.7	1.4	16.2
	P^Q4	0.2	2.0	0.2	3.7	0.3	5.8	0.4	8.8	0.3	12.7	0.2	16.6
	P^B7	0.6	2.4	0.9	4.1	1.2	6.1	1.4	9.0	1.3	12.7	0.9	16.3
	P^Q7	−0.1	1.9	−0.1	3.6	−0.1	5.7	0.0	8.8	−0.1	12.9	0.0	16.7
4	P^F	3.5	5.7	4.7	8.0	5.6	10.1	6.0	12.4	5.1	14.8	1.3	16.8
	P^χ2	−0.1	1.8	−0.5	3.2	−1.3	5.1	−3.1	8.1	−6.3	13.1	−11.4	20.3
	P^D	1.4	3.9	2.1	6.0	2.6	8.3	2.8	11.4	2.3	15.0	−0.1	18.4
	P^MD	1.4	3.9	2.1	6.1	2.7	8.4	2.9	11.3	2.1	14.7	−1.0	17.3
	P^BY	3.2	5.4	4.8	7.9	5.7	9.8	5.4	11.6	4.5	15.2	2.2	16.8
	P^M	2.2	4.9	3.4	7.5	4.5	10.1	5.4	13.4	5.7	16.9	4.1	19.2
	P^R	3.6	6.5	5.4	9.5	7.0	12.4	8.6	15.7	9.4	18.8	7.5	19.5
	P^T	0.5	3.1	0.8	5.3	1.0	7.9	1.3	11.6	1.3	16.3	0.8	20.5
	P^B4	1.4	3.8	2.0	6.1	2.6	8.5	3.0	11.8	2.8	15.8	1.2	19.3
	P^Q4	0.2	2.8	0.4	5.0	0.5	7.6	0.6	11.3	0.5	16.1	0.0	20.7
	P^B7	0.9	3.4	1.4	5.6	1.8	8.1	2.1	11.6	2.0	15.9	0.8	19.8
	P^Q7	−0.1	2.6	−0.1	4.9	0.0	7.6	0.0	11.5	0.1	16.5	0.0	21.0
8	P^F	5.6	8.9	7.3	11.6	8.5	14.0	9.0	16.4	7.5	18.4	2.1	19.7
	P^χ2	−0.6	1.4	−1.5	2.8	−3.1	4.9	−6.3	8.7	−12.3	15.7	−22.2	27.4
	P^D	2.4	6.1	3.4	8.9	4.2	11.8	4.6	15.2	3.8	19.1	0.4	22.6
	P^MD	2.4	6.1	3.4	8.9	4.2	11.7	4.5	14.8	3.2	17.8	−1.5	20.2
	P^BY	5.7	9.3	7.4	11.7	8.2	13.8	8.4	16.0	7.1	18.4	2.8	19.2
	P^M	3.6	7.9	5.3	11.2	6.8	14.5	8.1	18.1	8.2	21.7	5.5	23.7
	P^R	5.7	10.5	8.2	14.4	10.4	17.9	12.5	21.7	13.3	24.7	10.6	24.8
	P^T	1.0	5.1	1.6	8.2	2.3	11.6	2.8	16.1	3.0	21.6	2.0	26.3
	P^B4	2.2	6.0	3.2	8.9	4.1	11.9	4.6	15.6	4.2	19.9	1.3	23.4
	P^Q4	0.5	4.4	0.8	7.3	1.1	10.6	1.2	15.0	0.8	20.6	−0.7	25.6
	P^B7	1.6	5.4	2.3	8.3	3.0	11.4	3.4	15.4	3.0	20.3	0.9	24.6
	P^Q7	0.1	4.2	0.2	7.2	0.4	10.8	0.5	15.6	0.6	22.0	0.4	27.7

**Table 3:** Bias and root mean square error (SE) of the proposed estimates of P at $\nu_{2}=80$.

$\nu_{1}$		1%		2.5%		5%		10%		20%		40%	
		Bias	SE	Bias	SE	Bias	SE	Bias	SE	Bias	SE	Bias	SE
2	P^F	0.5	1.2	0.8	2.0	1.0	3.1	1.2	4.5	1.0	6.4	0.3	8.1
	P^χ2	0.1	0.8	0.0	1.6	0.0	2.6	−0.3	4.2	−0.8	6.2	−1.4	8.3
	P^D	0.2	1.0	0.3	1.8	0.5	2.8	0.5	4.4	0.4	6.4	0.0	8.2
	P^MD	0.2	1.0	0.4	1.8	0.4	2.9	0.5	4.4	0.4	6.4	0.0	8.2
	P^BY	0.6	1.2	0.8	2.0	1.1	3.2	1.2	4.3	0.9	6.4	0.2	8.1
	P^M	0.3	1.1	0.5	1.9	0.8	3.0	1.0	4.6	1.1	6.6	0.9	8.3
	P^R	0.5	1.2	0.8	2.1	1.1	3.2	1.4	4.8	1.6	6.7	1.3	8.2
	P^T	0.0	0.9	0.0	1.7	0.0	2.8	0.1	4.4	0.0	6.5	0.0	8.4
	P^B4	0.2	0.9	0.3	1.8	0.4	2.8	0.4	4.4	0.4	6.5	0.3	8.3
	P^Q4	0.0	0.8	0.0	1.7	0.0	2.8	0.0	4.4	0.0	6.5	0.0	8.4
	P^B7	0.1	0.9	0.2	1.7	0.2	2.8	0.3	4.4	0.3	6.5	0.2	8.3
	P^Q7	0.0	0.8	0.0	1.7	0.0	2.8	0.0	4.4	0.0	6.5	0.0	8.4
	P^B10	0.1	0.9	0.1	1.7	0.2	2.8	0.2	4.4	0.2	6.5	0.1	8.3
	P^Q10	0.0	0.8	0.0	1.7	0.0	2.8	0.0	4.4	0.0	6.5	0.0	8.4
4	P^F	0.8	1.7	1.3	2.8	1.6	4.1	1.8	5.8	1.5	8.0	0.4	9.8
	P^χ2	0.0	1.0	−0.1	1.9	−0.4	3.1	−1.0	5.0	−2.0	7.7	−3.5	10.5
	P^D	0.4	1.3	0.6	2.4	0.7	3.7	0.8	5.5	0.6	8.0	−0.1	10.1
	P^MD	0.4	1.3	0.6	2.4	0.7	3.7	0.8	5.6	0.6	8.0	−0.1	9.9
	P^BY	0.8	1.7	1.2	2.8	1.6	4.1	1.6	5.8	1.3	7.8	0.4	9.9
	P^M	0.5	1.4	0.8	2.6	1.1	3.9	1.4	5.8	1.5	8.3	1.0	10.2
	P^R	0.7	1.6	1.1	2.8	1.5	4.2	2.0	6.1	2.2	8.5	1.7	10.2
	P^T	0.0	1.1	0.1	2.1	0.1	3.5	0.1	5.5	0.1	8.2	0.0	10.4
	P^B4	0.3	1.2	0.4	2.3	0.6	3.6	0.6	5.6	0.6	8.1	0.3	10.3
	P^Q4	0.0	1.1	0.0	2.1	0.0	3.5	0.0	5.5	0.0	8.2	0.0	10.4
	P^B7	0.2	1.2	0.3	2.2	0.4	3.6	0.4	5.5	0.4	8.1	0.1	10.3
	P^Q7	0.0	1.1	0.0	2.1	0.0	3.5	0.0	5.5	0.0	8.2	0.0	10.4
	P^B10	0.1	1.1	0.2	2.2	0.3	3.5	0.3	5.5	0.3	8.2	0.1	10.4
	P^Q10	0.0	1.1	0.0	2.1	0.0	3.5	0.0	5.5	0.0	8.2	0.0	10.4
8	P^F	1.4	2.6	2.0	4.1	2.6	5.7	2.9	7.8	2.6	10.3	0.6	12.3
	P^χ2	−0.2	1.1	−0.6	2.1	−1.3	3.6	−2.6	6.0	−4.9	9.9	−8.1	14.6
	P^D	0.6	1.9	0.9	3.3	1.2	5.0	1.4	7.3	1.1	10.3	0.0	13.0
	P^MD	0.6	1.9	0.9	3.3	1.2	5.0	1.4	7.3	1.1	10.3	−0.3	12.5
	P^BY	1.4	2.6	1.9	3.9	2.3	5.6	2.8	7.9	2.3	10.1	0.9	12.2
	P^M	0.8	2.1	1.3	3.6	1.7	5.4	2.1	7.8	2.2	10.7	1.4	13.1
	P^R	1.0	2.3	1.7	4.0	2.3	5.8	3.0	8.3	3.3	11.1	2.5	13.2
	P^T	0.1	1.5	0.2	2.9	0.2	4.6	0.3	7.2	0.3	10.7	0.1	13.7
	P^B4	0.5	1.8	0.7	3.2	0.9	4.9	1.1	7.3	0.9	10.5	0.3	13.3
	P^Q4	0.0	1.5	0.0	2.8	0.0	4.6	0.0	7.2	0.0	10.7	0.0	13.7
	P^B7	0.3	1.6	0.5	3.0	0.6	4.7	0.7	7.2	0.6	10.5	0.1	13.5
	P^Q7	0.0	1.4	0.0	2.8	0.0	4.6	0.0	7.2	0.0	10.7	0.0	13.8
	P^B10	0.2	1.6	0.4	3.0	0.5	4.7	0.5	7.2	0.4	10.6	0.1	13.6
	P^Q10	0.0	1.4	0.0	2.8	0.0	4.6	0.0	7.2	0.0	10.7	0.0	13.8

**Table 4:** Median error (ME) and average absolute error (AAE) of the median estimates of P at $\nu_{1}=8$ and $\nu_{2}=40$.

	1%		2.5%		5%		40%	
	ME	AAE	ME	AAE	ME	AAE	ME	AAE
P^F	1.6	3.0	2.3	4.6	2.9	6.2	1.1	13.3
P^χ2	−0.8	0.9	−1.9	2.1	−3.6	3.7	−16.0	17.8
P^D	0.0	1.8	0.0	3.3	0.0	5.1	0.0	14.5
P^MD	0.0	1.8	0.0	3.3	0.0	5.1	0.0	13.7
P^BY	1.4	2.8	2.2	4.8	2.9	6.5	1.1	13.2
P^M	0.3	2.2	0.6	3.9	1.0	5.8	3.0	14.9
P^R	0.8	2.7	1.4	4.6	2.3	6.7	5.6	15.1
P^T	−0.7	1.4	−1.3	2.9	−2.0	4.9	0.4	16.1
P^B4	−0.1	1.7	−0.3	3.2	−0.4	5.0	0.4	15.1
P^Q4	−0.9	1.3	−1.7	2.9	−2.5	4.8	−0.3	16.2
P^B7	−0.4	1.5	−0.8	3.0	−1.1	4.9	0.1	15.5
P^Q7	−1.0	1.3	−1.9	2.8	−2.8	4.9	−0.1	16.4
P^B10	−0.5	1.5	−1.1	2.9	−1.5	4.9	0.0	15.8
P^Q10	−0.9	1.3	−1.8	2.8	−2.8	4.8	−0.1	16.4

**Table 5:** Median error (ME) and average absolute error (AAE) of the median estimates P^D and P^MD of P.

$\nu_{2}$	$\nu_{1}$		1%		2.5%		5%		40%	
			ME	AAE	ME	AAE	ME	AAE	ME	AAE
10	2	P^D	0.0	2.5	0.0	4.2	0.0	6.3	−0.1	17.1
	P^MD	0.0	2.5	0.0	4.2	0.0	6.3	−0.1	16.1
	4	P^D	0.0	3.6	0.0	5.8	0.0	8.3	0.0	20.2
	P^MD	0.0	3.6	0.0	5.8	0.0	8.2	0.0	17.8
20	2	P^D	0.0	1.6	0.0	2.8	0.0	4.4	0.0	12.6
	P^MD	0.0	1.6	0.0	2.8	0.0	4.4	0.0	12.3
	4	P^D	0.0	2.1	0.0	3.7	0.0	5.7	0.0	15.3
	P^MD	0.0	2.1	0.0	3.7	0.0	5.7	0.0	14.3
	8	P^D	0.0	3.1	0.0	5.2	−0.1	7.6	−0.1	19.1
	P^MD	0.0	3.1	0.0	5.2	−0.1	7.5	−0.1	17.1
80	2	P^D	0.0	0.7	0.0	1.3	0.0	2.2	0.1	6.6
	P^MD	0.0	0.7	0.0	1.3	0.0	2.2	0.1	6.6
	4	P^D	0.0	0.9	0.0	1.7	0.0	2.8	0.0	8.2
	P^MD	0.0	0.9	0.0	1.7	0.0	2.8	0.0	8.1
	8	P^D	0.0	1.2	0.0	2.2	0.0	3.6	0.0	10.6
	P^MD	0.0	1.2	0.0	2.2	0.0	3.6	0.0	10.2
